# Topical imiquimod adverse event: Beyond the local skin reaction—A case report

**DOI:** 10.1177/2050313X241274916

**Published:** 2024-08-19

**Authors:** Florence Lagacé-Thomassin, Ariana Nateghi, Elio Kechichian, Mylène Veilleux

**Affiliations:** Department of Dermatology, University of Sherbrooke, Sherbrooke, QC, Canada

**Keywords:** Case report, cutaneous skin reactions, adverse reactions, severe cutaneous adverse reactions, Stevens–Johnson syndrome, toxic epidermal necrolysis, basal cell carcinoma, imiquimod

## Abstract

Imiquimod is a well-known topical treatment for its efficacy against various skin conditions. While generally well-tolerated, adverse reactions like local skin irritation are common. However, severe systemic effects such as Stevens–Johnson syndrome (SJS) are rare, but possible. We present the case of an 82-year-old male who developed SJS following topical Imiquimod therapy for basal cell carcinoma. Despite minimal systemic absorption, serious reactions can occur, warranting caution. Prompt recognition and discontinuation of treatment are crucial for managing such rare but severe adverse events. This case underscores the importance of informed consent and vigilant monitoring for adverse reactions associated with Imiquimod therapy.

Imiquimod is a widely used topical treatment for condyloma acuminata, actinic keratosis, and basal cell carcinoma.^1^ When applied, Imiquimod activates the immune system through Toll-like receptor-7, leading to increased cytokine production and a resulting potent antitumor and antiviral effect.^1^ The well-known and expected effects of Imiquimod include local skin reactions such as erythema, crusting, scaling, ulceration, localized burning, and pruritus. Although generally well-tolerated, vitiligo, psoriasis, erythema multiforme, and Stevens–Johnson syndrome (SJS) have been reported as adverse events.^2^ Only two cases of Imiquimod-induced SJS have been reported previously to our knowledge.^3,4^ Systemic absorption is minimal; however, serious systemic effects can occur, as illustrated by the possibility of developing erythema multiforme and SJS.^1^ We herein report the case of SJS induced by topical Imiquimod application.

An 82-year-old male, with diabetes, hypertension, and dyslipidemia, presented to the emergency with oral pain and burning sensation, hindering food intake. These symptoms emerged 8 days postinitiation of topical Imiquimod therapy for left shoulder basal cell carcinoma. On physical exam, hemorrhagic crusts on the vermillion border, erosive gingivitis, stomatitis, and palate ulcers were noted (Figure 1). The complete skin exam revealed erosions on the application site as well as distant erosions on the back, covering 5% of the body surface along with atypical target lesions on the left arm and a positive Nikolsky sign (Figure 2). No lesions were noted on the ocular, genital, or anal mucosal surfaces. The patient had a history of using Imiquimod without incident and had no recent changes to his regular medication regimen. There were no known allergies.

**Figure 1.**
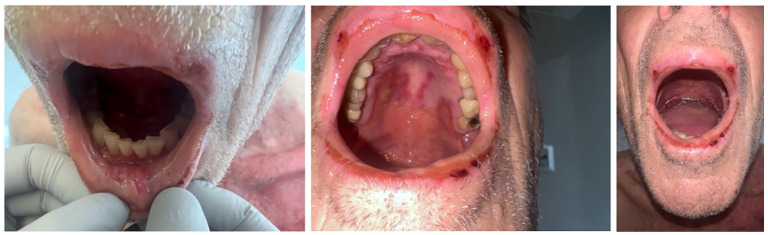
Oral mucosal involvement.

**Figure 2.**
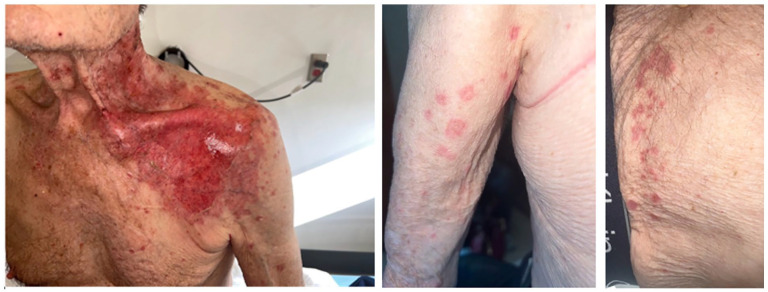
Cutaneous involvement: positive Nikolsky sign and atypical target lesions.

complete blood count, and chest X-ray were within normal limits. The differential diagnosis was erythema multiforme major versus SJS. Oral prednisone and valacyclovir were started but rapidly discontinued upon negative HSV/VZV PCR obtained from mucosal lesions as well as negative mycoplasma and Chlamydia pneumoniae serologies. The CRP was 17.8. The diagnosis of SJS was made. A skin biopsy of a flat target lesion on the back showed a complete skin ulceration without significant inflammation, and direct immunofluorescence was negative.

Following 48 h of Imiquimod cessation, along with supportive care and a soft diet, symptoms began to resolve, with no new lesions observed.

This case highlights the importance of recognizing a rare, yet severe side effect associated with topical Imiquimod, a commonly prescribed medication. SJS was probably induced by a delayed hypersensitivity reaction or by a systemic absorption of the drug with direct drug-induced necrolysis at distant sites. In clinical practice, it is essential to discuss potential adverse effects during informed consent for both medical and legal purposes. Prompt discontinuation of treatment, accompanied with thorough patient education about the risks linked to the development of mucosal lesions or distant skin lesions, is associated with a favorable prognosis.^5^ Despite its atypical presentation, SJS/toxic epidermal necrolysis should not be underestimated, even with repeated use of the medication. Physicians should be aware of Imiquimod’s side effects, emphasizing the importance of regular follow-up care. Diligent reporting of adverse drug events is not only encouraged but also vital in identifying further instances.
